# Identifying module biomarker in type 2 diabetes mellitus by discriminative area of functional activity

**DOI:** 10.1186/s12859-015-0519-y

**Published:** 2015-03-18

**Authors:** Xindong Zhang, Lin Gao, Zhi-Ping Liu, Luonan Chen

**Affiliations:** 1 School of Computer Science and Technology, Xidian University, Xi’an, 710000 China; 2 Key Laboratory of Systems Biology, Institute of Biochemistry and Cell Biology, Shanghai Institutes for Biological Sciences, Chinese Academy of Sciences, Shanghai, 200031 China; 3 Department of Biomedical Engineering, School of Control Science and Engineering, Shandong University, Shandong, 250061 China; 4 Institute of Industrial Science, University of Tokyo, Tokyo, 153-8505 Japan; 5 School of Life Science and Technology, ShanghaiTech University, Shanghai, 201210 China

**Keywords:** Computational biology, Gene expression profiling, Network biomarker, Type 2 diabetes mellitus

## Abstract

**Background:**

Identifying diagnosis and prognosis biomarkers from expression profiling data is of great significance for achieving personalized medicine and designing therapeutic strategy in complex diseases. However, the reproducibility of identified biomarkers across tissues and experiments is still a challenge for this issue.

**Results:**

We propose a strategy based on discriminative area of module activities to identify gene biomarkers which interconnect as a subnetwork or module by integrating gene expression data and protein-protein interactions. Then, we implement the procedure in T2DM as a case study and identify a module biomarker with 32 genes from mRNA expression data in skeletal muscle for T2DM. This module biomarker is enriched with known causal genes and related functions of T2DM. Further analysis shows that the module biomarker is of superior performance in classification, and has consistently high accuracies across tissues and experiments.

**Conclusion:**

The proposed approach can efficiently identify robust and functionally meaningful module biomarkers in T2DM, and could be employed in biomarker discovery of other complex diseases characterized by expression profiles.

**Electronic supplementary material:**

The online version of this article (doi:10.1186/s12859-015-0519-y) contains supplementary material, which is available to authorized users.

## Background

Type 2 diabetes mellitus (T2DM) formerly known as non-insulin dependent diabetes mellitus (NIDDM) or adult-onset diabetes is the most markedly growing chronic disease mainly caused by impairment in insulin secretion and insulin action [1]. A total of 285 million of people were estimated to suffer from T2DM in 2010 and would be doubled by 2030 [2,3]. Both environmental factors like lifestyle, obesity, poor diet, stress, nutritional status and genetic factors like genetic variations account for the development of T2DM [4]. In pathophysiology, insufficient insulin production in the setting of insulin resistance and inadequate insulin secretion in beta cell are two key features of T2DM [5], and lots of genetic variations are thought to contribute to the abnormal changes, and increase the risk of T2DM [6-11].

Discovery of gene biomarkers for complex diseases such as T2DM and various types of cancer is of great importance for prognosis, diagnosis, and the design of personalized medicine as well as therapeutic strategy. Researchers have proposed various methods to counter this issue, and lots of biomarkers have been identified to discriminate patients with different disease subtypes or different clinical prognosis, which are helpful for effective treatment in the last decade [12-15]. Often, these biomarkers cannot capture substrate relationships between phenotypes and genotypes, thus provide little information in pathogenesis of diseases. On the other hand, with recent rapid advance of modern high-throughput technologies, massive amounts of omics data have been used to cater for this need. Biomarkers extracted from these types of data not only provide new insights in prognosis of disease states or subtypes, but also a better understanding of the pathogenesis of complex diseases [16].

However, low reproducibility across experiments or tissues with the difficulty to gain a clear biological interpretation still exists for the ignorance of the systematic context gene functions, which can be modeled as a biological network, such as protein-protein interactions (PPI) and regulatory networks. A more effective means to address this difficulty is to integrate information from molecular interaction networks. Protein-protein interaction is considered to be an important way to facilitate biological functions, such as DNA replication and signal transduction which play fundamental roles in many biological processes. Using PPI networks derived from literature and databases, a number of approaches have demonstrated the effectiveness to identify discriminative modules, or so-called network biomarkers to various diseases [17-22].

On the other hand, discovering gene biomarkers from gene expression data is also of great importance in prognosis, understanding the mechanisms of T2DM and designing personalized medicine and therapeutic strategy. In this work, a novel method is proposed to identify a set of interacted genes with discriminant ability from gene expression data, which are defined as “module biomarker”. The proposed method is applied to identify module biomarker for T2DM by integrating gene expression profiling data and PPI interactions. It is well known that skeletal muscle in the dominant position of insulin-mediated uptake plays an important role in the pathogenesis of insulin resistance and is responsible for more than 80% of insulin-stimulated whole body glucose disposal. It is considered that skeletal muscle insulin resistance is the primary defect in T2DM [23]. Thus study in skeletal muscle is of great significance in extracting meaningful biomarkers of T2DM. In this method, we first generate a group of discriminative modules by optimizing the discriminative ability of module activities, and then a priori knowledge-based method is used to select the potentially robust module biomarker. Finally, a robust and stable module biomarker of 32 genes for T2DM is identified and further validates by various independent datasets. The identified module biomarker is functionally meaningful and enriched with T2DM related pathways and diseases genes. Interestingly, we find that few of these disease genes are differentially expressed across tissues, but they are highly interconnected to form a subnetwork in the PPI network (PPIN) and play a central role in the module biomarker by interconnecting differentially expressed genes.

## Results and discussion

### Overview

**Figure 1:**
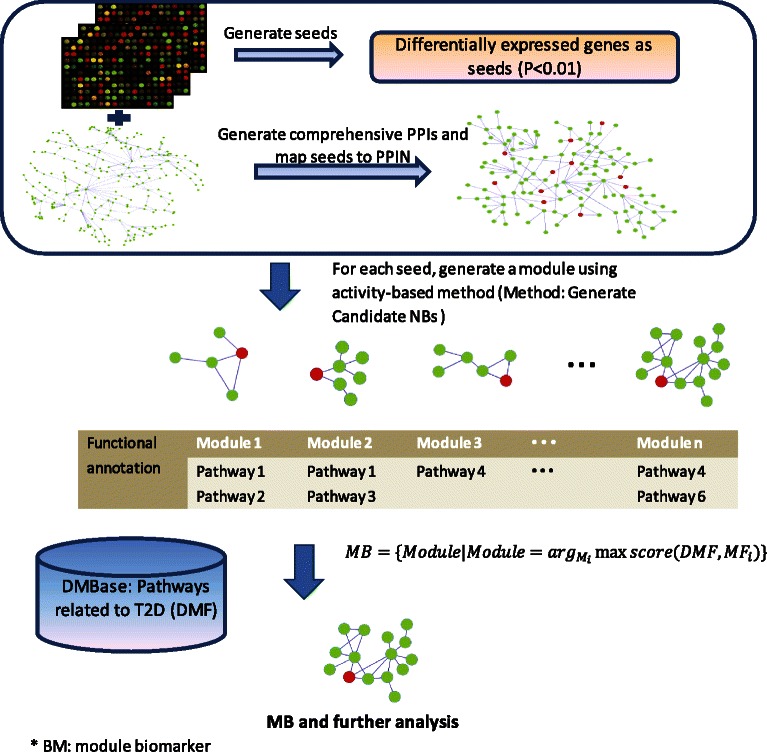
**Overview of the proposed framework for identifying module biomarker.**

Figure 1 shows the flowchart of our method for identifying module biomarker. The main idea is that genes function as modules, and the activity of group of genes or modules may be enhanced or weakened by their interactors.

In this work, we hypothesize that the activity of genes or modules following normal distribution under specific conditions. This assumption has been applied to pathway activity-based classification [24]. According to this assumption, we model activities of a module under two conditions (e.g., normal *vs.* case) following normal distributions with parameters *μ*_*N*_, *σ*_*N*_ and *μ*_*C*_, *σ*_*C*_ respectively. Then the common area under the two distribution curves is determined by given *μ*_*N*_, *σ*_*N*_ and *μ*_*C*_, *σ*_*C*_, and is defined as discriminative area. Clearly, the smaller the overlapped size of the discriminative area, the larger the difference between the two distributions. Thus, the purpose of this work is to find a set of genes which satisfy

1. these genes interact as a module in the background network,
2. the activity of the module is of the smallest discriminative area, and
3. genes in the set can be served as gene biomarkers with robust performance in discriminating whether a given sample is contained in the normal or case group.

**Figure 2:**
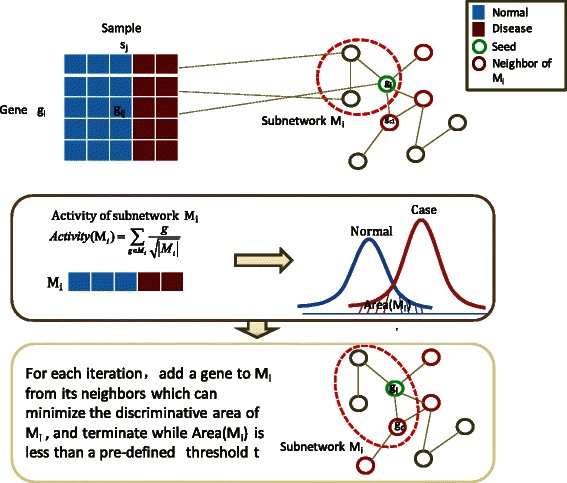
**Computational strategy for generating discriminative modules.** Computational strategy for generating discriminative modules by maximizing discriminative area of module activity. The discriminative area is defined as the area under two probability density functions of module activities corresponding to normal samples and case (disease) samples.

**Figure 3:**
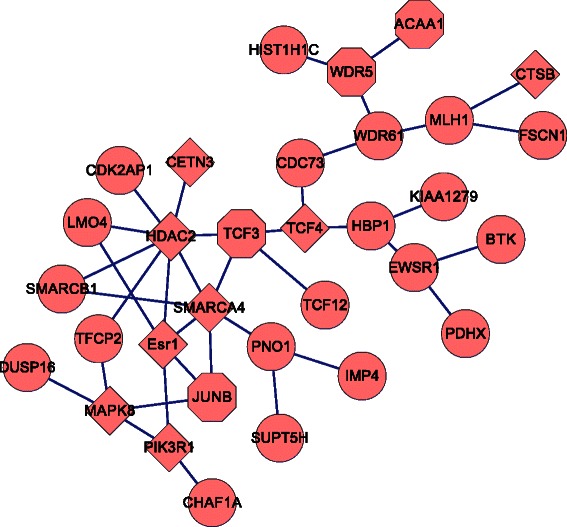
**Network structure of identified module.** Network structure of identified module which contains 32 genes, where diamond denotes that the gene is a causal gene of T2DM by quering T2D-Db or GAD, hexagon denotes that the gene is a T2DM related gene by functional correlation.

To capture significant changes of genes in transcriptional expression level, we first identified 203 differentially expressed genes as seeds with adjusted *p*-value <0.01 by *t*-test, and then generated a discriminative module for each seed by a greedy strategy. Figure 2 shows the main idea of the seed-growth strategy (see Methods for details). Hence, by removing modules of discriminative area *disa* (M) >0.2, 40 modules remained after selection. The activities of these 40 modules are highly correlated *PCC* >0.6, which indicates that these modules have a poor effect on improving discriminative ability, and each of them could be regarded as a potential module biomarker for the original data (GSE18732). Then, we used a function-similarity based method to detect a module which would be more reproducible across data sets. Finally, a module of 32 genes with the highest score was identified. Figure 3 shows interactions of these 32 genes in module biomarker, and Additional file 1: Table S1 for the details of these 32 genes.

### Validations of module biomarker

We investigated the classification performance of the identified module biomarker by a number of independent gene expression datasets across tissues. As the result shown, the identified module biomarker has a superior classification performance and has consistently high accuracies across tissues and datasets.

We tested whether 32 genes in the identified module can be served as biomarkers for type 2 diabetes mellitus in different expression datasets (GSE18732, E-MEXP-2559, GSE20966, GSE23343, and GSE26887). All these datasets refer to different experiments and tissues (see Methods). Gene profiles of these 32 genes in the module biomarker as features to model classifier by a SVM with linear kernel function in these datasets and 10-fold cross-validation was employed to evaluate classification accuracy. The result shows that the identified module biomarker of 32 genes not only have a high classification accuracy in skeletal muscle profiles (92.39% for GSE18732 and 80% for E-MEXP-2559), but also in beta cell (80% for GSE20966), liver (88.24% for GSE23343) and left ventricle (83.33% for GSE26887), which means these 32 genes have a superior classification accuracy across tissues and experiments.

**Figure 4:**
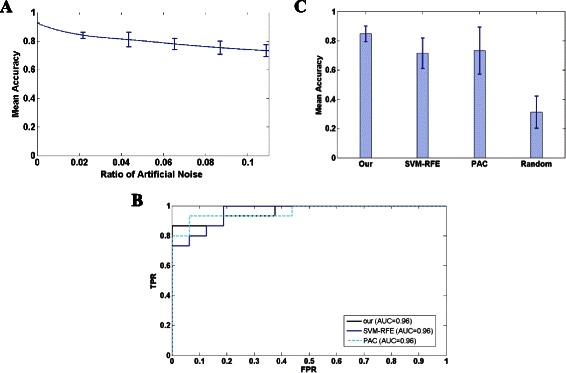
**Performance analysis of the identified module biomarker. (A)** The robustness of classification accuracy in perturbation data with different ratio of artificial noises. The mean accuracy of the proposed classifier decreases progressively from 84.02% to 73.26% when ratio of noise increases from 1% to 10%. **(B)** Comparison of biomarkers identified by different methods in GSE18732. ROC curves shows a superior performance in classification of module biomarker identified in this work (AUC = 0.96). **(C)** Histogram of mean accuracy with variance for biomarkers identified by our method, SVM-RFE and PAC. We also randomized the interactions of background network (PPIs) 50 times and identified a module biomarker using the proposed method, then mean accuracy and variance are calculated for 10-fold cross-validation across 5 datasets used in this work. Results show a stable performance across tissues for identified biomarkers.

For avoiding over-fitting of classifier, we employed 10-fold cross-validation and randomly changed certain percentage of class attributes as artificial noise by 100 times in training dataset. The confidence interval was used to measure correlations between artificial noises and classification accuracies. We used GSE18732 as a case study for enough instances. The result shows that the identified module biomarker maintains a relatively high mean accuracy when the percentage of artificial noise increases from 1% to 10%, which implies the robustness of the classifier induced by identified module biomarker (Figure 4A).

Then we compared the module biomarker identified in this work with biomarkers identified by two well-known methods, SVM-RFE [25] and PAC [24] in dataset GSE18732. SVM-RFE conducts feature selection in a recursive elimination manner, and was initially proposed for binary classification. PAC summarizes the pathway activity level by extracting its condition responsive genes (CORGs). Finally, 720 genes and 10 pathways identified by SVM-RFE and PAC were selected for further study respectively. The dataset was divided into training set (31 normal *vs.* 30 case), and test set (16 normal vs. 15 case). The SVM with linear kernel was applied to generate classifiers. As a result, biomarkers identified in this work obtained a predictive accuracy 87.09% with AUC 0.96 and prediction accuracy 90.32% with AUC 0.96 for SVM-RFE, 87.10% with AUC 0.96 for PAC. Figure 4B shows the ROC curves of these three biomarkers in predicting test instances. Then we performed a 10-fold cross-validation in all five dataset (GSE18732, E-MEXP-2559, GSE20966, GSE23343, and GSE26887) to these three biomarkers (Table 1). Although the highest predictive accuracy, the mean accuracy for the module biomarker identified in this work is more stable across tissues (Figure 4C).

**Table 1:** **Accuracy of different biomarkers across experiments by 10-fold cross-validation**

**Biomarkers**	**Dataset**					**Mean ± Variance**
	**GSE18732**	**E-MEXP-2559**	**GSE20966**	**GSE23343**	**GSE26887**	
**Module biomarker (32 genes)**	**92.39%**	80%	80%	**88.24%**	83.33%	**84.79% ± 0.054**
**SVM-RFE**	67.39%	80%	85%	58.82%	75%	**73.24% ± 0.103**
**PAC**	84.78%	75%	60%	47.06%	83.33%	**70.03% ± 0.16**
32 top differentially expressed genes	73.91%	75%	75%	82.35%	**100%**	81.25% ± 0.11
Type 2 diabetes mellitus	55.43%	80%	90%	35.29%	91%	70.34% ± 0.24
B cell receptor signalling pathway	60.87%	**85%**	**95%**	70.59%	83.33%	78.96% ± 0.133
Toll like receptor signalling pathway	48.91%	70%	80%	**88.24%**	83.33%	74.1% ± 0.156
Biosynthesis of unsaturated fatty acids	55.43%	80%	65%	**88.24%**	75%	72.73% ± 0.128
Insulin signalling pathway	59.78%	**85%**	85%	58.82%	**100%**	77.72% ± 0.179

The best results for nine obtained biomarkers in each dataset are shown in boldface.

We also selected top 32 differentially expressed genes and other five T2DM-related pathways (type 2 diabetes mellitus, B cell receptor signalling pathway, toll like receptor signalling pathway, biosynthesis of unsaturated fatty acids, insulin signalling pathway) as matched biomarkers. The *p*-value of genes was calculated by a *t*-test method and genes of adjusted *p*-value < 0.01 were considered to be differentially expressed. Five pathways were selected from the background pathway set and functionally enriched in module biomarker. We compared the classification performance of the identified module biomarker to differentially expressed gene biomarkers and pathway-based biomarkers on all 5 datasets. Table 1 shows accuracies of these biomarkers in all five datasets.

We found that the classification accuracy of our module biomarker is consistently high in all datasets (92.39% in GSE18732, 80% in E-MEXP-2559, 80% GSE20966, 88.24% in GSE23343, 83.33% in GSE26887). On the other hand, we noticed that the classification accuracy of our module biomarker is not always the maximal one, differentially expressed gene biomarkers and pathway-based biomarkers can also obtain high classification accuracies in some datasets. For example, the classification accuracies of differentially expressed genes and insulin signalling pathway even reach 100% in GSE26887, 90% for type 2 diabetes in GSE20966, while 83.33% and 88.24% for our module biomarker in GSE26887 and GSE20966 respectively. We then compared the stability of all these biomarkers by mean classification accuracies and variances across all datasets. The mean accuracy and standard variation of our module biomarker is 84.79% ± 0.054, while 81.25% ± 0.11 for differentially expressed gene biomarkers, 70.34% ± 0.24 for type 2 diabetes mellitus, 78.96% ± 0.133 for B cell receptor signalling pathway, 74.1% ± 0.156 for toll like receptor signalling pathway, 72.73% ± 0.128 for biosynthesis of unsaturated fatty acids, and 77.72% ± 0.179 for insulin signalling pathway. The result shows that our module biomarker has the highest mean classification performance and the lowest standard variance, which implies that our module biomarker is more stable than differentially expressed gene biomarkers and pathway-based biomarkers. The high accuracy of classification also provides evidence for the discriminative power of biomarker property underlying the identified module biomarker.

### Module-based biomarker analysis

#### Functional implications

Performing database query in T2D-Db [26] and GAD [27], we found that eight genes, i.e., CETN3, CTSB, ESR1, HDAC2, MAPK8, PIK3R1, SMARCA4, TCF4 are documented disease genes of T2DM. Of these, 7 genes (ESR1, HDAC2, MAPK8, PIK3R1, SMARCA4, TCF4 and CETN3) highly interact. The interactions of these 7 genes were shown in Additional file 1: Figure S1.

Besides 8 known disease genes, many genes also have a relationship to T2DM by literature mining or play a role in pathways associated to T2DM [28-35]. For instances, ACAA, TCF3, JUNB and WDR5. ACAA1 is a key gene involved in lipid oxidation and glucose metabolism, both of which are highly related to T2DM [28,29]. TCF3 is a transcriptional factor involved in the initiation of neuronal differentiation, and plays a role in muscle cell differentiation and cell development. Heterodimers between TCF3 and tissue-specific basic helix-loop-helix (bHLH) proteins play major roles in determining tissue-specific cell fate during embryogenesis, like muscle or early B-cell differentiation (function annotation of TCF3 in UniprotKB) [230]. A recent study has suggested that low muscle mass associated with type II diabetes risk [31,32]. JUNB is also a transcriptional factor which is involved in regulating gene activity following the primary growth factor response. It maintains skeletal muscle mass and promotes hypertrophy [33]. WDR5 has an effect on the molecular regulation of myogenesis by cooperating with Ash2L and MLL2 to form a histone methyltransferase (HMT) complex, which is recruited by Pax7 factor to remodel the chromatin structure for the control of the muscle lineage-specific gene expression [34,35].

We then extracted enriched pathways of module biomarker in KEGG [36,37] using a hypergeometric test, and the p-value is adjusted by Benjamini-Hochberg method [38]. Results indicate that the module biomarker is enriched with T2DM related pathways such as Type 2 diabetes mellitus (*p* <10^− 3^), B cell receptor signalling pathway (p <0.003), Insulin signalling pathway (p <0.013), Toll like receptor signalling pathway (p <0.006), and Biosynthesis of unsaturated fatty acid (p <0.036) (full list can be found in Table 2).

**Table 2:** **Enriched KEGG pathways of biomarker module**

**KEGG Pathway**	**Corrected P-value**
Fc epsilon RI signaling pathway	0.000177920
Type 2 diabetes mellitus	0.000616943
B cell receptor signaling pathway	0.002456893
Progesterone mediated oocyte maturation	0.003642362
ERBB signaling pathway	0.003764635
Toll like receptor signalling pathway	0.005905897
Biosynthesis of unsaturated fatty acids	0.036209447
Mismatch repair	0.002893819
Neurotrophin signaling pathway	0.010609564
Insulin signaling pathway	0.013322982

#### Tissue-specific module biomarker

We then investigated gene activities in identified module biomarker in different tissues, and discovered the relationships among tissue specific differentially expressed genes, T2DM related genes and identified module biomarker.

The module biomarker has different sets of differentially expressed genes in different tissues, such as TCF12, MAPK8, MLH1, LMO4, CDC73, HIST1H1C, WDR61, WDR5 in GSE18732, SUPT5H, TCF3 and WDR5 in E-MEXP-2995 (skeletal muscle), CTSB, TCF4, LMO4 and HBP1 in GSE20966 (beta-cells from pancreatic tissue), CETN3, PIK3R1, SMARCB1, CDK2AP1, LMO4, WDR5, PNO1, CDC73 and WDR61 in GSE23343 (liver), BTK, CTSB, JUNB, FSCN1 and TCF4 in GSE26887 (left ventricle (LV) cardiac biopsies). Among these tissue-specific differentially expressed gene sets, few T2DM related genes are differentially expressed by *t*-test with p-value less than 0.05 (skeletal muscle (MAPK8, WDR5 in GSE18732), Beta cell from pancreatic tissue (CTSB, TCF4 in GSE20966), Liver (CTEN3, PIK3R1, WDR5 in GSE23343), left ventricle (LV) cardiac biopsies (CTSB, TCF4, JUNB in GSE26887)). And also, few overlaps share among these tissue-specific differentially expressed gene sets. Interestingly, we found that all tissue-specific differentially expressed genes in the module biomarker tightly interact to T2DM related genes which also highly interconnect in PPIN (see Additional file 1: Figure S1-S6 for network construction between T2DM related genes and tissue-specific differentially expressed genes in Additional file 1).

We also tested the classification performance of these tissue specific differentially expressed genes in 5 independent datasets, and the result shows that these tissue specific differentially expressed genes have high classification accuracy across tissues (77.17% for GSE18732, 80% for E-MEXP-2995, 85% for GSE20966, 94.12% for GSE23343 and 100% for GSE26887), which indicates that the identified module biomarker has specific gene activities in different datasets corresponding to different tissues. However, these tissue-specific gene actions differ from tissue to tissue and implies poor reproducible classification performance across tissues.

Although few overlaps among tissue-specific differentially expressed genes and poor reproducibility across tissues, the module biomarker shows strong stability in classification performance across tissues for capturing relationships between tissue-specific gene actions and T2DM related genes, which may reveal potential pathological mechanisms for T2DM.

## Conclusions

We propose a novel module-based method to identify network biomarkers for T2DM on skeletal muscle. A module biomarker with 32 genes is identified. The module biomarker is more accuracy in classification performance than traditional biomarkers, i.e., gene-based biomarkers and pathway-based biomarkers, and also has consistently high classification accuracy when applied in different tissues. The module biomarker is enriched with T2DM related genes and T2DM related pathways, which implies that the module biomarker is functionally meaningful. 32 genes in module biomarker are also enriched with causal genes of T2DM. In particular, 4 genes, ACAA1, TCF3, JUNB and WDR5, are functionally related genes for T2DM by a literature analysis, and play major roles in muscle mass and regulate important actions of hypertrophy, and can be served as candidate disease genes for T2DM. All 8 causal genes and 4 T2DM related genes directly interacted to form a module. Analysis of module biomarkers in specific tissues indicates that the module biomarker can capture relationships of tissue specific differentially expressed genes and T2DM related genes, which may reveal potential pathological mechanisms for T2DM, and makes the module biomarker more stable across tissues.

## Methods

### Datasets

All datasets used in this work were downloaded from public data portals. We downloaded the gene expression data (GSE18732) for T2DM from Gene Expression Omnibus (GEO) [39], which consists of mRNA extracted from skeletal muscle of 47 normal (NGT) subjects, 26 glucose intolerant (IGT) subjects and 45 type 2 diabetic (DM) subjects. The expression data were normalized by z-score, and only NGT and DM subjects were selected in this work. For a given gene g, let *X* = (*x*_*1*_, *x*_*2*_ ⋯, *x*_*n*_) be the expression vector of g across *n* instances, the z-score can be calculated as follows,

$$ Z\left(\mathrm{g}\right)=\frac{\overline{x}-X\;}{\sigma }, $$

where $\overline{x}$ is the mean of *X* and *σ* is the standard deviation of *X*.

We also downloaded other datasets from GEO and ArrayExpress [40] as independent datasets referred to different experiments and tissues. E-MEXP-2559 [41] was downloaded from ArrayExpress. This dataset contains 5 normal subjects, 15 first degree relatives, 5 type 2 diabetic subjects. All subjects were Caucasian males and biopsies were taken after a controlled metabolic period of a two hour hyperinsulinemic euglycemic clamp. GSE20966 contains 10 control and 10 type 2 diabetic subjects obtained from beta-cells from pancreatic tissue sections by the laser capture microdissection technique [42]. GSE23343 contains 10 patients with type 2 diabetes and 7 subjects with normal glucose tolerance from hepatic tissues with percutaneous needle liver biopsy [43]. GSE26887 contains 7 T2DM heart failure patients, 12 non-T2DM heart failure patients and 5 controls from left ventricle (LV) cardiac biopsies [44]. Only control/normal subjects and DM subjects were selected for further study in this work.

The protein-protein interaction network was downloaded from iRefIndex (version 9.0) [45], which integrate multiple types of interactions (physical and genetic) from a number of primary interaction databases (BIND [46], BioGRID [47], CORUM [48], DIP [49], HPRD [50], IntAct [51], MINT [52], MPact [53], MPPI [54] and OPHID [55]). The iRefIndex consists of 32475 interactors and 401140 interactions. We filtered PPINs using gene expression data, genes both in PPINs and microarray were used in the following analysis. Thus, the final PPIN contains 8028 interactors/gene products and 58253 interactions.

The background T2DM related pathways were collected from Genetic Database for Diabetes Mellitus (DMBase) [56].

### Methods

Figure 1 shows the flow chart of our method for identifying subnetwork or module biomarker, which is described in this section.

### Seed selection

Differentially expressed genes can capture significant changes of genes in transcription level between different conditions. So we calculate P-value for each genes in PPIN, and 190 genes with adjusted *p*-value <0.01 are selected as seeds.

### Identification of discriminative modules

We use a greedy strategy to generate a module of maximal discriminative ability for each seed. Figure 2 shows the flowchart of this process. These modules are defined as discriminative modules.

Our method is based on the assumption that the activity of a group of genes or module is normal distribution. This assumption has been discussed above. For a given module *M* corresponding to seed g,the activity vector of *M* is

$$ a\left(\mathrm{M}\right)={\displaystyle \sum_{g_i\in M}\frac{a\left({g}_i\right)}{\sqrt{k}}} $$

where *a*(g_*i*_) denotes the expression vector of *g*_*i*_, *k* is the size of *M*. We define the discriminative area of (*M*(*disa*(M))) as the area under probability density functions (PDFs) of *a*(M) corresponding to control and disease states. Then the greedy strategy is

$$ \begin{array}{l}\frac{ \min }{g_c} disa\left(a\left(\mathrm{M}\mathrm{U}\left\{{\mathrm{g}}_c\right\}\right)\right)\\ {} subject\kern0.62em to\kern0.62em {g}_c\kern0.5em \in N\left(\mathrm{M}\right)\\ {}N\left(\mathrm{M}\right)={\displaystyle \underset{i=1}{\overset{k}{\cup }}N\left({g}_i\right)},\kern0.5em {g}_i\in M\end{array} $$ where *N*(*g*) denotes the neighbour set of gene g in PPIN. The iteration is terminated if *disa*(M) is less than a predefined threshold *δ* (in this work *δ* = 0.001).

### Network biomarker selection

As the strategy opted, discriminative modules highly fit the original expression data but not all of them can be regard as biomarker. Thus we used a functional similarity-based method to evaluate these modules. We collected 19 T2DM related pathways from DMBase as a background set (see Additional file 1: Table S2 in for full descriptions of these 19 pathways).

Then we scored each discriminative module as the similarity between the enriched pathways (MF) and background set (DMF). We used a hypergeometric test to access whether a pathway P is in KEGG and a module M

$$ p=1-{\displaystyle \sum_{i=0}^{s-1}\frac{\left(\begin{array}{c}\hfill {n}_2\hfill \\ {}\hfill i\hfill \end{array}\right)\left(\begin{array}{c}\hfill n-{n}_2\hfill \\ {}\hfill {n}_1-i\hfill \end{array}\right)}{\left(\begin{array}{c}\hfill n\hfill \\ {}\hfill {n}_1\hfill \end{array}\right)}} $$

where *n* is the total number of nodes in PPIN. *n*_1_ and *PS*_1_ = {ps_11_, ps_12_, ⋯, ps_1*m*_} are the sizes of P and M, respectively. The similarity of two pathway sets can be calculated as follows [57]:

$$ sim\left({\mathrm{PS}}_1,{\mathrm{PS}}_2\right)=\frac{{\displaystyle \sum_{1\le i\le m}sim\left({\mathrm{ps}}_{1i},{\mathrm{PS}}_2\right)}+{\displaystyle \sum_{1\le j\le n}sim\left({\mathrm{ps}}_{2j},{\mathrm{PS}}_1\right)}}{m+n} $$

where *PS*_1_ = {ps_11_, ps_12_, ⋯, ps_1*m*_} and *PS*_2_ = {ps_21_, ps_22_, ⋯, ps_2*n*_} denote two pathway sets. *ps* is the gene set of a pathway and

$$ sim\left(\mathrm{ps},\mathrm{P}\mathrm{S}\right)=\underset{1<i<k}{ \max}\kern0.5em sim\kern0.20em \left({\mathrm{ps},\mathrm{ps}}_i\right) $$

$$ sim\left({\mathrm{ps}}_1,{\mathrm{ps}}_2\right)=\frac{\left|p{s}_1{\displaystyle \cap\;p{s}_2}\right|}{\left|p{s}_1{\displaystyle \cup p{s}_2}\right|} $$

where *k* is the size of *ps*_*i*_.

## Additional file

Additional file 1:
**This document provides detailed descriptions of context not included in the paper.** **Table S1.** Detailed description of 32 genes in identified module biomarker. **Table S2.** 19 T2DM related pathways downloaded from DMBase used in the paper. **Figure S1-S6.** Connections of causal genes and tissue specific differentially expressed genes in different datasets across tissues.
