# Supplementary material for: Identifying module biomarker in type 2 diabetes mellitus by discriminative area of functional activity
Source: BMC Bioinformatics. 2015 Mar 18;16:92. doi: 10.1186/s12859-015-0519-y (PMC4374500; doi:10.1186/s12859-015-0519-y)
Supplement: Additional file 1: — This document provides detailed descriptions of context not included in the paper. Table S1. Detailed description of 32 genes in identified module biomarker. Table S2. 19 T2DM related pathways downloaded from DMBase used in the paper. Figure S1-S6. Connections of causal genes and tissue specific differentially expressed genes in different datasets across tissues. [file 12859_2015_519_MOESM1_ESM.doc]

**Supplementary files**

**Table S1. List of 32 genes in Biomarker module**

| Gene symbol | Description |
| --- | --- |
| ACAA1 | Acethl-Coenzyme A acyltransferase 1 |
| BTK | Bruton agammaglobulinemia tyrosine kinase |
| CDC73 | Cell division cycle 73, Paf1/RNA polymerase II complex component |
| CDK2AP1 | Cyclin-dependent kinase 2 associated protein 1 |
| CETN3 | Centrin, EF-hand protein, 3 |
| CHAF1A | Chromatin assembly factor 1, subunit A |
| CTSB | Cathepsin B |
| DUSP16 | Dual specificity phosphatase 16 |
| ESR1 | Estrogen receptor 1 |
| EWSR1 | Similar to Ewing sarcoma breakpoint region 1; Ewing sarcoma breakpoint region 1 |
| FSCN1 | Fascin homolog 1, actin-bundling protein |
| HBP1 | HMG-box transcription factor 1 |
| HDAC2 | Histone deacetylase 2 |
| HIST1H1C | Histone cluster 1, H1C |
| IMP4 | IMP4, U3 small nucleolar ribonucleoprotein |
| JUNB | Jun B proto-oncogene |
| KIAA1279 | KIAA1279 |
| LMO4 | LIM domain only 4 |
| MAPK8 | Mitogen-activated protein kinase 8 |
| MLH1 | MutL homolog 1, colon cancer, nonpolyposis type 2 (E. coli) |
| PDHX | Pyruvate dehydrogenase complex, component X |
| PIK3R1 | Phosphoinositide-3-kinase, regulatory subunit 1 (alpha) |
| PNO1 | Partner of NOB1 |
| SMARCA4 | SWI/SNF related, matrix associated, actin dependent regulator of chromatin, subfamily a, member 4 |
| SMARCB1 | SWI/SNF related, matrix associated, actin dependent regulator of chromatin, subfamily b, member 1 |
| SUPT5H | Suppressor of Ty 5 |
| TCF12 | Transcription factor 12 |
| TCF3 | Transcription factor 3 (E2A immunoglobulin enhancer binding factors E12/E47) |
| TCF4 | Transcription factor 4 |
| TFCP2 | Transcription factor CP2 |
| WDR5 | WD repeat domain 5 |
| WDR61 | WD repeat domain 61 |

Table S2. 19 T2DM related pathways in DMBase

| **No.** | **Pathway (Database)** | **Description** | **Number of genes** |
| --- | --- | --- | --- |
| 1 | ADIPOCYTOKINE SIGNALING PATHWAY (KEGG) | Adipocytokine signaling pathway | 67 |
| 2 | TYPE II DIABETES MELLITUS (KEGG) | Type II diabetes mellitus | 47 |
| 3 | INSULIN SIGNALING PATHWAY (KEGG) | Insulin signaling pathway | 137 |
| 4 | MATURITY ONSET DIABETES OF THE YOUNG (KEGG) | Maturity onset diabetes of the young | 25 |
| 5 | GH PATHWAY (BIOCARTA) | Growth Hormone Signaling Pathway | 28 |
| 6 | SIGNAL ATTENUATION (REACTOME) | Genes involved in Signal attenuation | 11 |
| 7 | ALDOSTERONE REGULATED SODIUM REABSORPTION (KEGG) | Aldosterone-regulated sodium reabsorption | 42 |
| 8 | INSULIN PATHWAY (BIOCARTA) | Insulin Signaling Pathway | 22 |
| 9 | PPAR SIGNALING PATHWAY (KEGG) | PPAR signaling pathway | 69 |
| 10 | TYPE I DIABETES MELLITUS (KEGG) | Type I diabetes mellitus | 44 |
| 11 | FACILITATIVE NA INDEPENDENT GLUCOSE TRANSPORTERS (REACTOME) | Genes involved in Facilitative Na+-independent glucose transporters | 12 |
| 12 | IGF1 PATHWAY (BIOCARTA) | IGF-1 Signaling Pathway | 21 |
| 13 | G ALPHA S SIGNALLING EVENTS (REACTOME) | Genes involved in G alpha (s) signaling events | 124 |
| 14 | IL1R PATHWAY (BIOCARTA) | Signal transduction through IL1R | 33 |
| 15 | REGULATION OF INSULIN SECRETION BY GLUCAGON LIKE PEPTIDE 1 (REACTOME) | Genes involved in Regulation of Insulin Secretion by Glucagon-like Peptide-1 | 61 |
| 16 | SHC RELATED EVENTS (REACTOME) | Genes involved in SHC-related events | 14 |
| 17 | ERYTH PATHWAY (BIOCARTA) | Erythrocyte Differentiation Pathway | 15 |
| 18 | LONGEVITY PATHWAY (BIOCARTA) | The IGF-1 Receptor and Longevity | 15 |
| 19 | HORMONE BIOSYNTHESIS (REACTOME) | Genes involved in Hormone biosynthesis | 52 |

Figure S1-S6: The connection of T2D related genes and tissue specific differentially expressed genes in different datasets across different tissues, where diamond denotes that the gene is a causal gene of T2DM in database, hexagon denotes that the gene is a T2DM related gene by functional correlation. The green color denotes that the gene is a differentially expressed genes. The result shows that biomarker module identified has a tissue specific action in different datasets. Of these, 8 genes are causal genes of T2DM reported in datasets, 4 genes are T2DM functionally related genes. T2DM related genes directly interact with each other, which implies that the biomarker module is functionally meaningful.


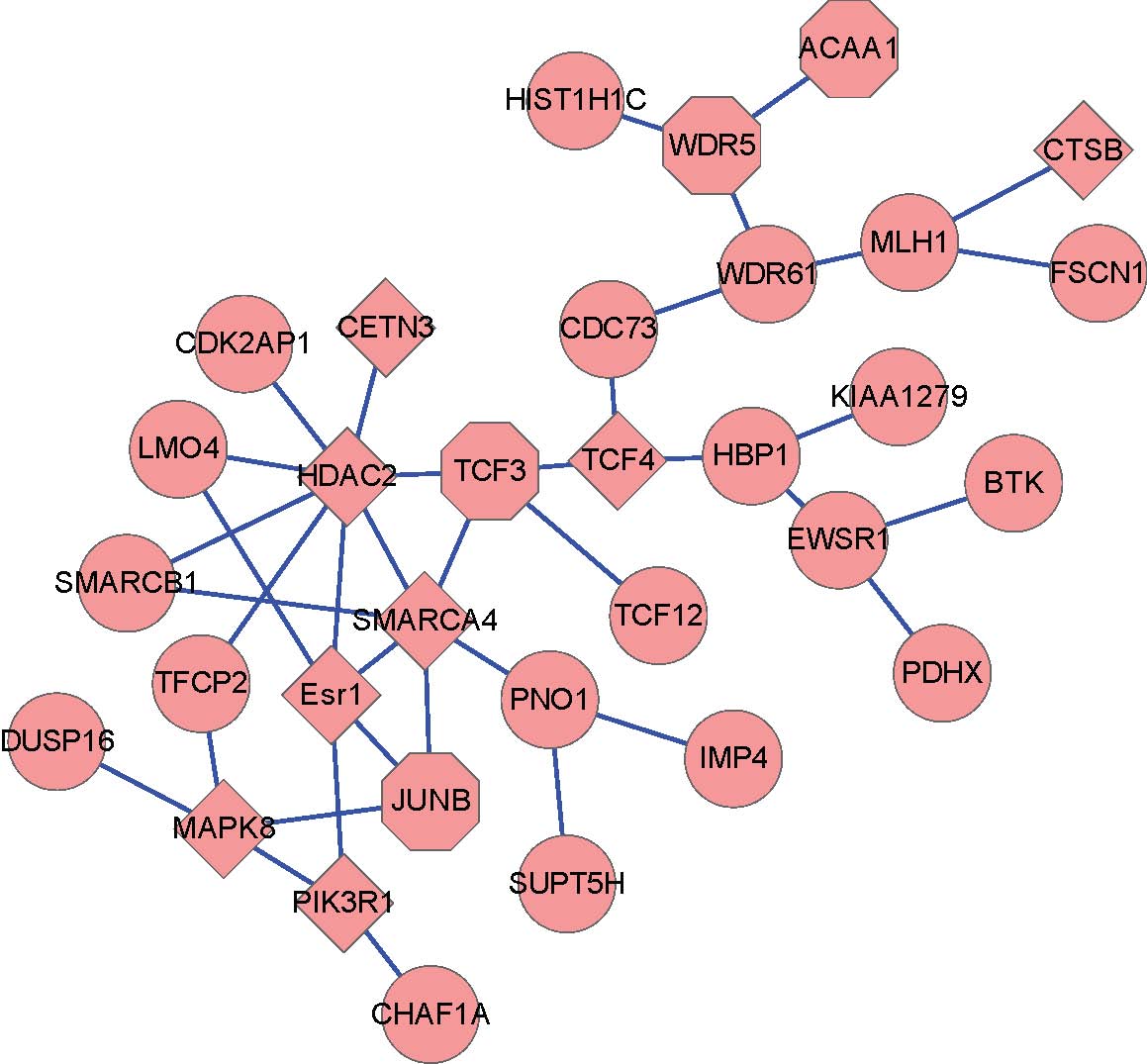


Figure S1 Network structure for identified module biomarker of 32 genes


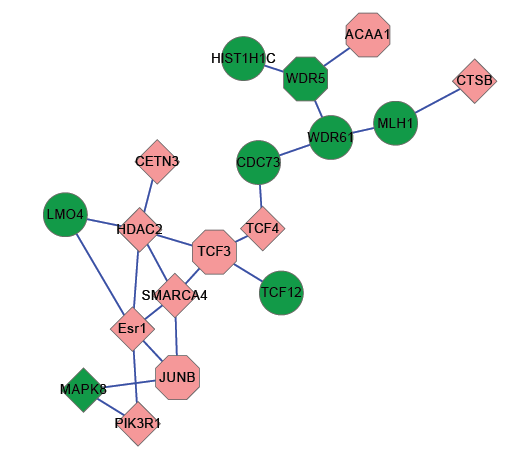


Figure S2. Interactions of T2D related genes and tissue specific differentially expressed genes in skeletal muscle (GSE18732)


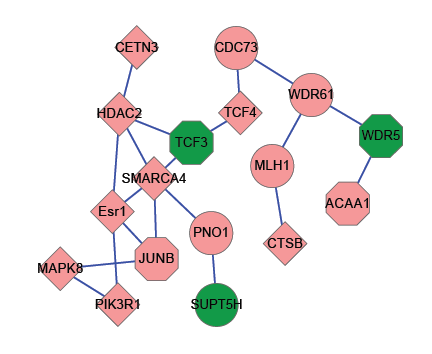


Figure S3. Interactions of T2D related genes and tissue specific differentially expressed genes in skeletal muscle (E-MEXP-2995)


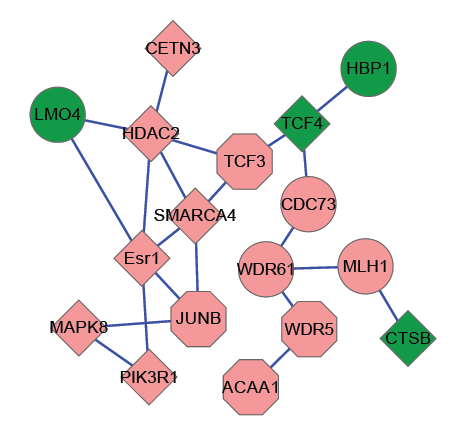


Figure S4. Interactions of T2D related genes and tissue specific differentially expressed genes in beta cell (GSE20966)


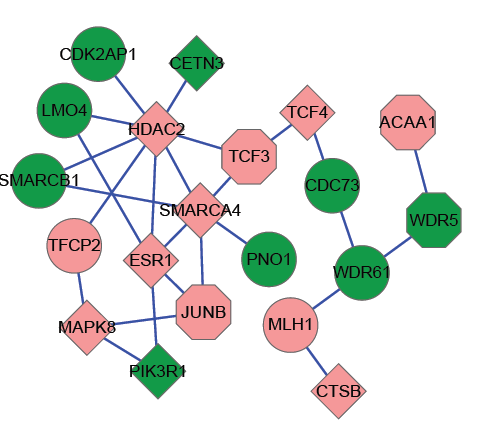


Figure S5. Interactions of T2D related genes and tissue specific differentially expressed genes in liver(GSE23343)


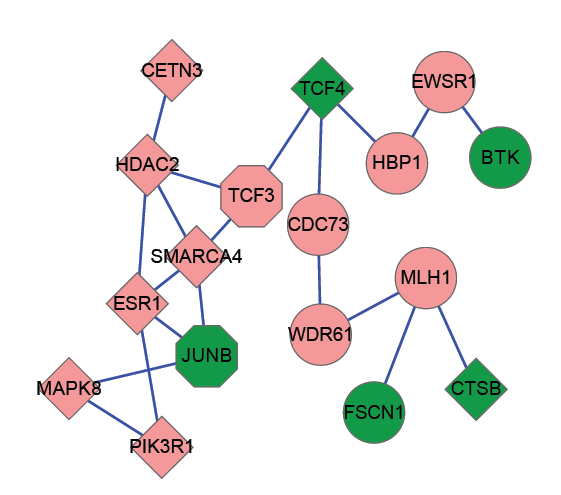


Figure S6. Interactions of T2D related genes and tissue specific differentially expressed genes in left ventricle (GSE26887)
